# Defective ciliogenesis in thyroid hürthle cell tumors is associated with increased autophagy

**DOI:** 10.18632/oncotarget.12997

**Published:** 2016-10-31

**Authors:** Junguee Lee, Shinae Yi, Yea Eun Kang, Joon Young Chang, Jung Tae Kim, Hae Joung Sul, Jong Ok Kim, Jin Man Kim, Joon Kim, Anna Maria Porcelli, Koon Soon Kim, Minho Shong

**Affiliations:** ^1^ Department of Pathology, Daejeon St. Mary's Hospital, College of Medicine, The Catholic University of Korea, Jung-gu, Daejeon, 34943, Republic of Korea; ^2^ Research Center for Endocrine and Metabolic Diseases, Division of Endocrinology, Department of Internal Medicine, Chungnam National University School of Medicine, Jung-gu, Daejeon 35015, Republic of Korea; ^3^ Department of Medical Science, Chungnam National University School of Medicine, Jung-gu, Daejeon 35015, Republic of Korea; ^4^ Department of Pathology, Chungnam National University School of Medicine, Jung-gu, Daejeon 35015, Republic of Korea; ^5^ Graduate School of Medical Science and Engineering, KAIST, Yuseong-gu, Daejeon 34141, Republic of Korea; ^6^ Department of Pharmacy and Biotechnology-FABIT, University of Bologna, Bologna, Italy and Interdepartmental Industrial Research Center on Health Sciences and Technologies, University of Bologna, Bologna, Italy

**Keywords:** primary cilia, defective ciliogenesis, thyroid hürthle cell, autophagy

## Abstract

Primary cilia are found in the apical membrane of thyrocytes, where they may play a role in the maintenance of follicular homeostasis. In this study, we examined the distribution of primary cilia in the human thyroid cancer to address the involvement of abnormal ciliogenesis in different thyroid cancers. We examined 92 human thyroid tissues, including nodular hyperplasia, Hashimoto's thyroiditis, follicular tumor, Hürthle cell tumor, and papillary carcinoma to observe the distribution of primary cilia. The distribution and length of primary cilia facing the follicular lumen were uniform across variable-sized follicles in the normal thyroid gland. However, most Hürthle cells found in benign and malignant thyroid diseases were devoid of primary cilia. Conventional variant of papillary carcinoma (PTC) displayed longer primary cilia than those of healthy tissue, whereas both the frequency and length of primary cilia were decreased in oncocytic variant of PTC. In addition, ciliogenesis was markedly defective in primary Hürthle cell tumors, including Hürthle cell adenomas and carcinomas, which showed higher level of autophagosome biogenesis. Remarkably, inhibition of autophagosome formation by *Atg5* silencing or treatment with pharmacological inhibitors of autophagosome formation restored ciliogenesis in the Hürthle cell carcinoma cell line XTC.UC1 which exhibits a high basal autophagic flux. Moreover, the inhibition of autophagy promoted the accumulation of two factors critical for ciliogenesis, IFT88 and ARL13B. These results suggest that abnormal ciliogenesis, a common feature of Hürthle cells in diseased thyroid glands, is associated with increased basal autophagy.

## INTRODUCTION

Primary cilia in mammalian cells are crucial organelles for sensory reception and signal transduction, and their roles are closely linked with cell type-specific functions [1, 2]. In thyroid epithelial cells, primary cilia protrude from the apical surface into the follicular luminal space, which contains colloid. It is generally accepted that, in thyroid epithelial cells, primary cilia sense the environment of the follicular lumen and contribute to follicular function, including the production of hormones [3–5]. The maintenance of primary ciliary function in specific cell types requires highly regulated mechanisms of ciliogenesis, which, if altered, can lead to primary ciliopathy, a disease that may show clinical phenotypes of congenital hypothyroidism [6]. Ciliogenesis is tightly regulated by molecular programs that control the steps required for both the assembly of the axoneme and the biogenesis of the ciliary membrane [7]. The first step of ciliogenesis is the migration of the centriole-derived basal body to the cell surface where distal appendages of the basal body establish the link between the plasma membrane and the basal body. Thereafter, axonemal microtubules extend from the basal body through the process of intraflagellar transport (IFT), and docking of transport vesicles at the base of the growing cilia promotes ciliary membrane biogenesis [8].

Recently, it has been shown that ciliogenesis and autophagy are bidirectionally regulated [9–12]. Autophagy is a lysosome-dependent degradation process that removes cell constituents, cellular organelles, and protein aggregates [13]. Basal autophagy appears to prevent ciliary growth through the degradation of ciliary proteins such as IFT20, a key component in IFT [10]. By contrast, enhanced autophagy triggered by starvation stimulates the degradation of oral-facial-digital syndrome 1 (OFD1), the endogenous inhibitor of ciliogenesis, thereby promoting ciliogenesis [11]. However, abnormal activation of autophagy results in IFT20 degradation, which impedes the unlimited growth of the cilia [14]. On the other hand, the loss of primary cilia has been shown to partially inhibit autophagy [10].

Hürthle cells, which are found in chronic inflammatory and tumorigenic thyroid glands, are oncocytic cells with higher amounts of abnormal mitochondria, which results in an abundantly acidophilic, granular cytoplasm [13]. Increases in mitochondrial content are caused by the accumulation of damaged mitochondria possessing mitochondrial DNA (mtDNA) mutations in respiratory complex genes that cause severe bioenergetic crisis [15–18]. Recently, we found that XTC.UC1 cells derived from Hürthle cell tumors have higher levels of autophagosome formation [12]. However, it remains unclear whether this feature is linked to the cellular functions of Hürthle cells, which are biologically less active than those of normal epithelial cells [17, 19].

In this study, we examined the appearance of primary cilia in the human thyroid gland and the alternations in ciliogenesis in thyroid diseases, namely, Hashimoto's thyroiditis, follicular tumors, Hürthle cell tumors, and papillary thyroid carcinomas. Interestingly, primary cilia were abnormal in Hürthle cells in Hashimoto's thyroiditis, papillary thyroid cancer and primary Hürthle cell tumors. In addition, ciliogenesis was suppressed in the Hürthle cell line XTC.UC1, which shows a relatively high level of autophagic activity. We found that genetic and pharmacologic inhibition of autophagy turnover in XTC.UC1 cells promoted ciliogenesis as well as ciliary elongation. We propose that activated autophagy flux impedes ciliogenesis in Hürthle cells with compromised mitochondrial oxidative phosphorylation (OxPhos). The identification of the molecular mechanism underlying defective ciliogenesis in Hürthle cell tumors could help understand the clinical features of thyroid diseases.

## RESULTS

### Identification of primary cilia in normal human thyroid gland

**Figure 1:**
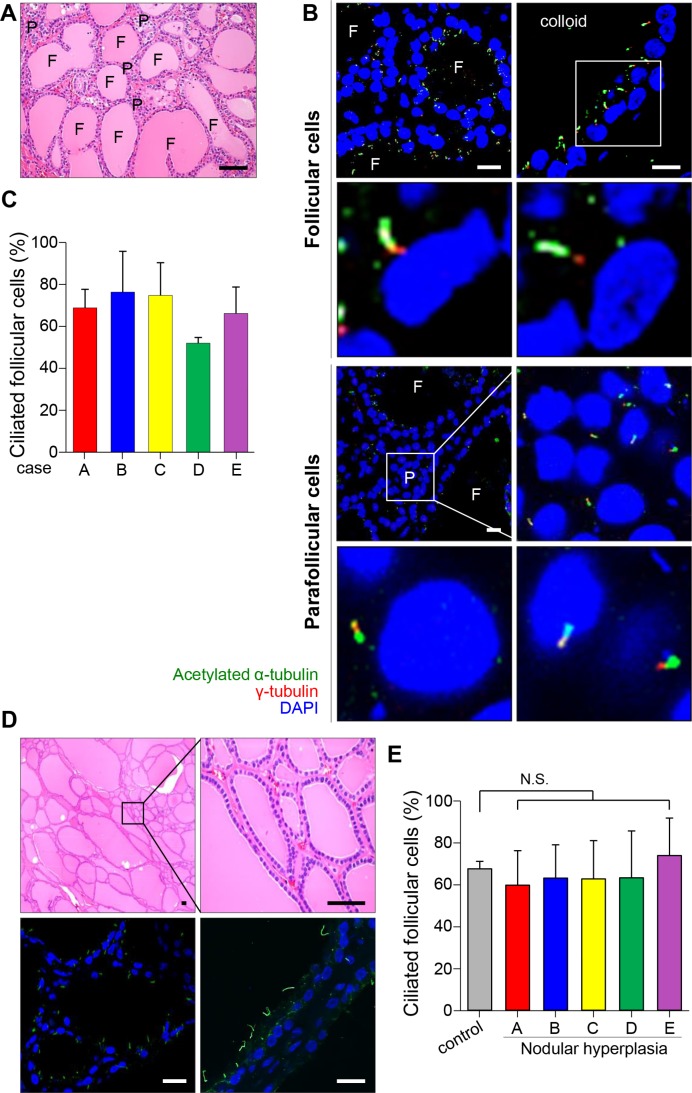
Distribution of primary cilia in thyroid tissue with normal and nodular hyperplasia (**A**) Normal thyroid stained with H&E. F, follicles; P, parafollicular cells. Scale bar, 30 μm. (**B**) Immunofluorescent staining of primary cilia in follicular and parafollicular cells of the normal thyroid using anti-acetylated α-tubulin (green) and anti-γ-tubulin (red). F, follicles; P, parafollicular cells. Scale bar, 5 μm. (**C**) The average frequency of primary cilia in the follicles of five cases with normal thyroid glands. (**D**) Nodular hyperplasia of thyroid stained with H&E. Scale bar, 20 μm. Immunofluorescent staining of primary cilia in the nodular hyperplasia using anti-acetylated α-tubulin (green) and anti-γ-tubulin (red). Scale bar, 5 μm. (**E**) The average frequency of primary cilia in follicles of five patients with nodular hyperplasia. Normal follicular cells were used as controls. N.S.; not significant.

Thyroid follicles lined with a single layer of epithelial cells are structural and functional units that produce thyroid hormone. We identified primary cilia using antibodies against acetylated α-tubulin and ARL13B, a cilia-enriched small GTPase. The staining of acetylated α -tubulin and Arl13B has been widely used for the determination of ciliated cell frequency and cilia length [20, 21]. The detection of basal bodies, the root of primary cilia, was performed by immunofluorescent staining using an antibody against γ-tubulin, which localized in the basal bodies. We assessed ciliated cell frequency in the normal follicular epithelium *in vivo* by immunofluorescent staining of five specimens taken from the contralateral lobe of thyroid cancer. The tissue cross-sections were stained with haematoxylin and eosin (H&E) to identify normal follicles (Figure 1A). As shown in Figure 1B, primary cilia were detected in both follicular epithelial cells and parafollicular cells. It has been reported that primary cilia usually extend from the apical surface of secretory cells [22]. As expected, primary cilia in follicular cells extended from the apical membrane toward the colloid-rich follicular lumen. More than 50% of the epithelial cells showed uniformly ciliated patterns in normal follicles (Figure 1C).

### Expression patterns of primary cilia in benign thyroid diseases

One of the representative thyroid diseases exhibiting follicular heterogeneity is benign nodular hyperplasia (NH), which shows structural variability in follicles (Figure 1D). No remarkable changes in either the frequency of ciliated cells or the lengths of cilia were found in benign nodular hyperplasia when they were compared with those in normal thyroid glands (normal 67.8 ± 3.6% vs NH 64.8 ± 18.3%, *p* = 0.363) (Figure 1D and 1E). This finding indicates that benign structural variability found in nodular hyperplasia does not associate with abnormalities in ciliogenesis.

**Figure 2:**
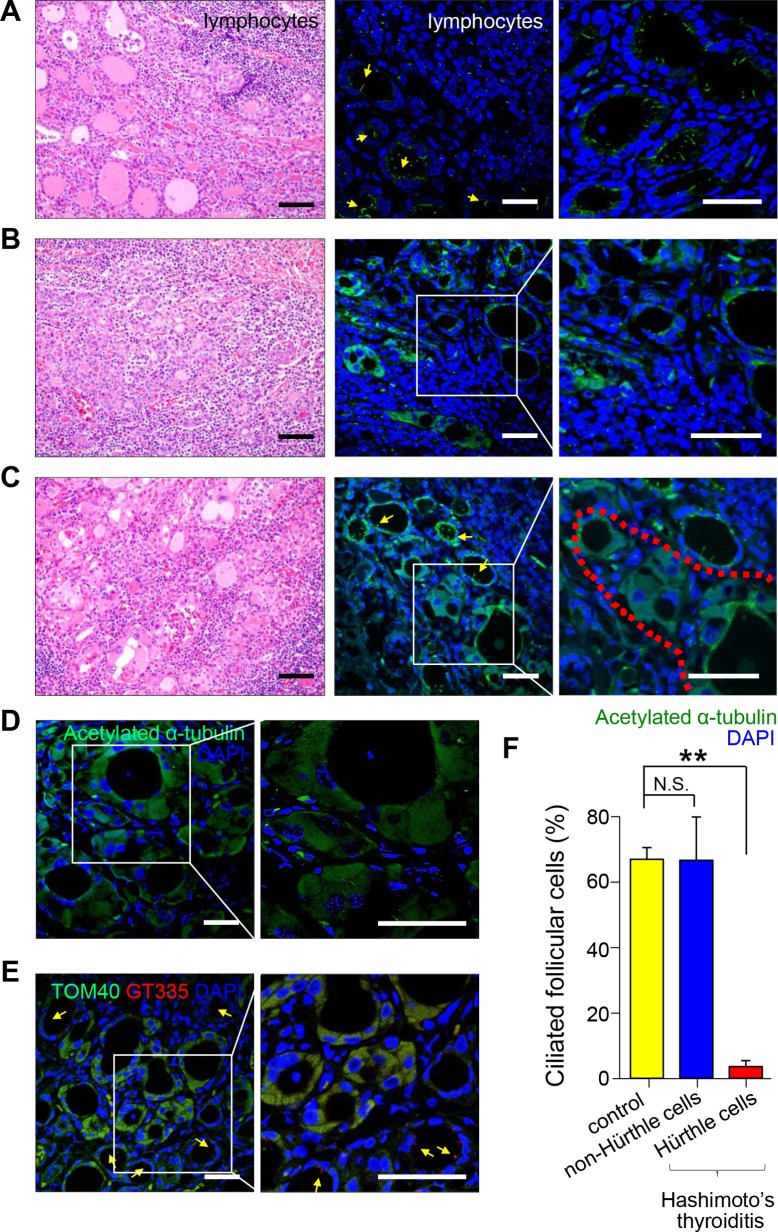
Distribution of primary cilia in Hashimoto's thyroiditis (**A**) Small and atrophic follicles with infiltrating lymphoplasma cells stained with H&E. Scale bar, 30 μm. Arrows indicate follicles. Immunofluorescent staining of primary cilia using anti-acetylated α-tubulin (green). Scale bar, 10 μm. (**B**) Follicles damaged by infiltrating lymphoplasma cells and fibrosis stained with H&E. Scale bar, 30 μm. Immunofluorescent staining of primary cilia using anti-acetylated α-tubulin (green). Scale bar, 10 μm. (**C**) Hürthle cells with abundant eosinophilic cytoplasm stained with H&E. Scale bar, 30 μm. The red dotted line indicates Hürthle cells. Immunofluorescent staining of primary cilia using anti-acetylated α-tubulin (green). Scale bar, 10 μm. (**D**) Immunostaining of rare primary cilia in Hürthle cells using anti-acetylated α-tubulin (green). Arrows indicate non-Hürthle cells with primary cilia. The square box indicates Hürthle cells. Scale bar, 10 μm. (**E**) Hürthle cells showed intense expression of mitochondrial proteins, TOM40 (green). Immunofluorescent staining of primary cilia using anti-GT335 (red). Scale bar, 10 μm. (**F**) The average frequency of primary cilia in non-Hürthle and Hürthle cells in the thyroid glands of patients with Hashimoto's thyroiditis. Normal follicular cells were used as controls. **p < 0.01, N.S.; not significant.

Hashimoto's thyroiditis (HT) is a representative chronic thyroiditis accompanied by variable degrees of follicular damage with heavy infiltration of immune cells into the stroma surrounding the thyroid follicles [23]. Follicles found in areas close to lymphocyte infiltrations were smaller and filled with scanty colloid (Figure 2A). These follicular epithelial cells showed normal features of the primary cilia, and the percentage of ciliated epithelial cells was similar to that of the normal thyroid gland (normal 67.8 ± 3.6% vs HT 67.5 ± 13.4%, *p* = 0.472) (Figure 2A and 2F). The follicles infiltrated with lymphocytes also showed primary cilia (Figure 2B). The atrophic follicles with abundant Hürthle cells were observed as isolated follicular structures (Figure 2C). Interestingly, Hürthle cells of Hashimoto's thyroiditis rarely displayed primary cilia (normal 67.8 ± 3.6% vs Hürthle cell of HT 3.6 ± 1.9%, *p* = 0.0007)(Figure 2C and 2F). The pathogenesis of Hürthle cells in Hashimoto's thyroiditis may be secondary to a mutation in mtDNA that causes mitochondrial dysfunction. The staining of Hürthle cells with an antibody against acetylated α–tubulin showed a diffuse distribution pattern in the cytoplasm, unlike normal thyroid cells (Figure 2D). a-Tubulin is an intrinsic mitochondrial structural protein required for molecular transport, and a significant portion of α–tubulin is acetylated in mitochondria [24]. Thus, excessive accumulation of mitochondria may cause increased immunoreactivity of acetylated α–tubulin in the cytoplasm. Translocase of outer mitochondrial membrane 40 (TOM40) was used as a marker of mitochondria density. Hürthle cells in Hashimoto's thyroiditis showed strong expression of TOM40 but showed decreased distribution of primary cilia (Figure 2E). Together, these results suggest that Hürthle cells have altered primary ciliogenesis that may be linked to defects in mitochondrial oxidative phosphorylation.

### Expression patterns of primary cilia in malignant thyroid tumors

**Figure 3:**
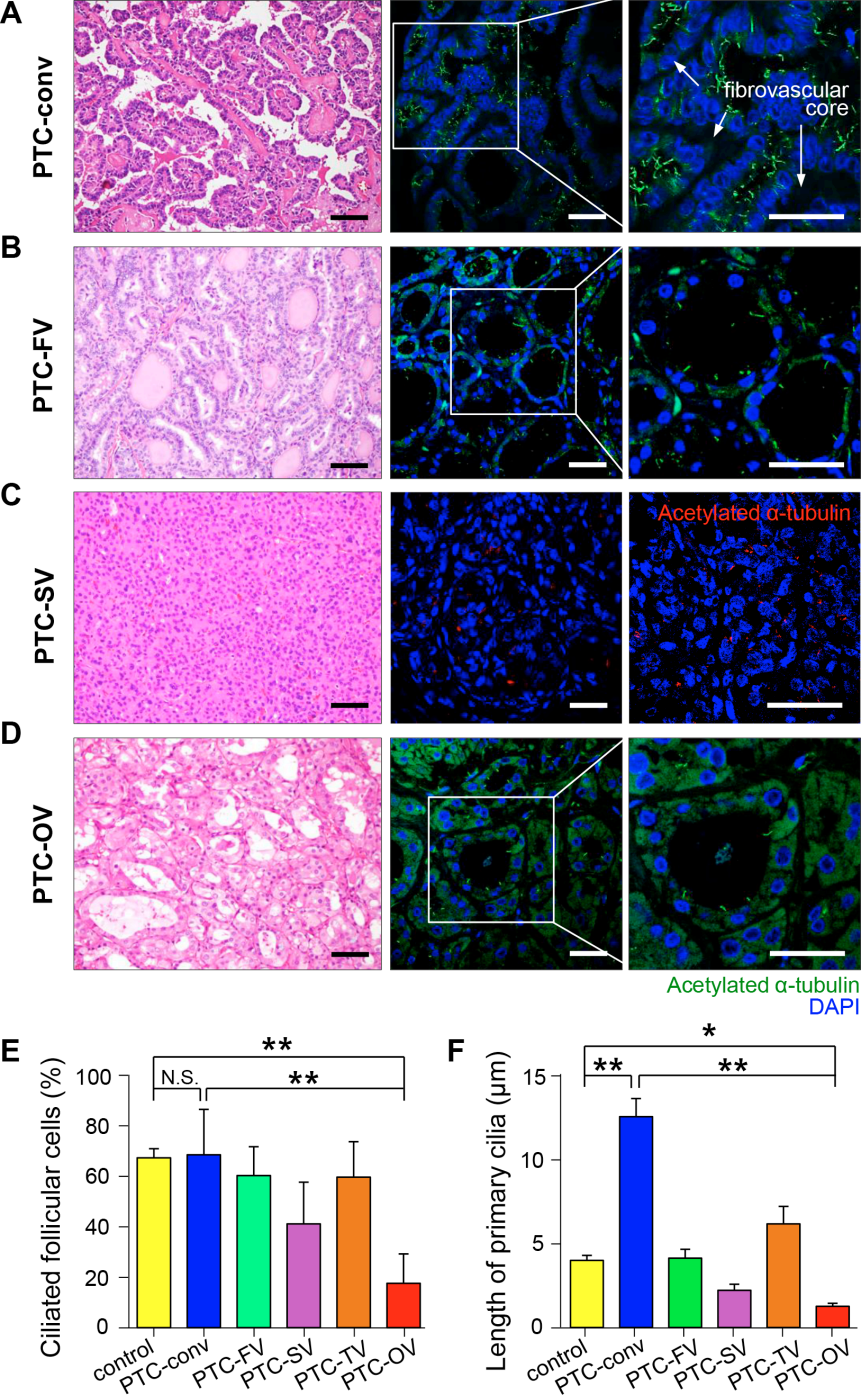
Distribution of primary cilia in papillary thyroid carcinoma (**A**) Conventional PTC (PTC-conv) composed of complex papillae with thin fibrovascular cores stained with H&E. Scale bar, 30 μm. Immunofluorescent staining of primary cilia using anti-acetylated α-tubulin (green). Scale bar, 10 μm. (**B**) Follicular variant of PTC (PTC-FV) showing follicular morphology with PTC nuclear features stained with H&E. Scale bar, 30 μm. Immunofluorescent staining of primary cilia using anti-acetylated α-tubulin (green). Scale bar, 10 μm. (**C**) Solid variant of PTC (PTC-SV) composed of round solid nests stained with H&E. Scale bar, 30 μm. Immunofluorescent staining of primary cilia using anti-acetylated α-tubulin (red). Scale bar, 10 μm. (**D**) Oncocytic variant of PTC (PTC-OV) composed of Hürthle cells with PTC nuclear features stained with H&E. Scale bar, 30 μm. Immunofluorescent staining of primary cilia using anti-acetylated α-tubulin (green). Scale bar, 10 μm. (**E**) The average frequency of primary cilia in different PTC variants compared with that of the normal thyroid. (**F**) The average length of primary cilia in different PTC variants compared with that of the normal thyroid. PTC-conv, conventional; PTC-FV, follicular variant; PTC-SV, solid variant; PTC-TV, tall cell variant; PTC-OV, oncocytic variant of papillary carcinoma. Normal follicular cells were used as controls. *p < 0.05, **p < 0.01, N.S.; not significant.

To further substantiate the finding of abnormal ciliogenesis in Hürthle cells in malignant thyroid diseases, we examined the distribution of primary cilia in papillary thyroid cancer (PTC). PTC is the most common type of thyroid cancer and has multiple histopathological variants, including conventional, follicular, oncocytic (Hürthle cell), solid, and tall cell variants [25]. We examined the expression of primary cilia in histopathological variants of PTC. The conventional PTC was characterized by complex papillae with thin fibrovascular cores, and the cancer cells in this PTC variant showed well-expressed primary cilia (Figure 3A). The other relatively common variant of PTC, follicular variant of PTC characterized by follicular architecture with PTC nuclear features, showed a similar expression pattern of primary cilia compared to that of the conventional type (Figure 3B). The frequency of ciliated cells was comparable in four different variant types, namely, conventional (conv), follicular variant (FV), solid variant (SV), tall cell variant (TC), and oncocytic variant (OV) (Figure 3E). However, the length of primary cilia showed characteristic alterations according to histopathological variants (Figure 3F). The conventional variant showed significantly longer primary cilia, whereas the solid variant showed shorter primary cilia compared to that of normal thyroid cells. Similar to Hürthle cells found in Hashimoto's thyroiditis, the oncocytic variant of PTC had remarkably fewer ciliated cells compared to normal follicular cells (normal 67.8 ± 3.6% vs PTC-OV 17.6 ± 11.7%, *p* = 0.0002) or the conventional type of PTC (PTC-conv 68.7 ± 18.0% vs PTC-OV 17.6 ± 11.7%, *p* = 0.0027). In addition, we observed increased diffuse cytoplasmic staining of anti-acetylated α–tubulin in the oncocytic variant, suggesting excessive accumulation of mitochondria (Figure 3D). Taken together, these findings suggest that ciliogenesis is influenced by the variant-specific pathogenesis of PTC, particularly the oncocytic variant of PTC.

### Defective ciliogenesis in Hürthle cell tumors

**Figure 4:**
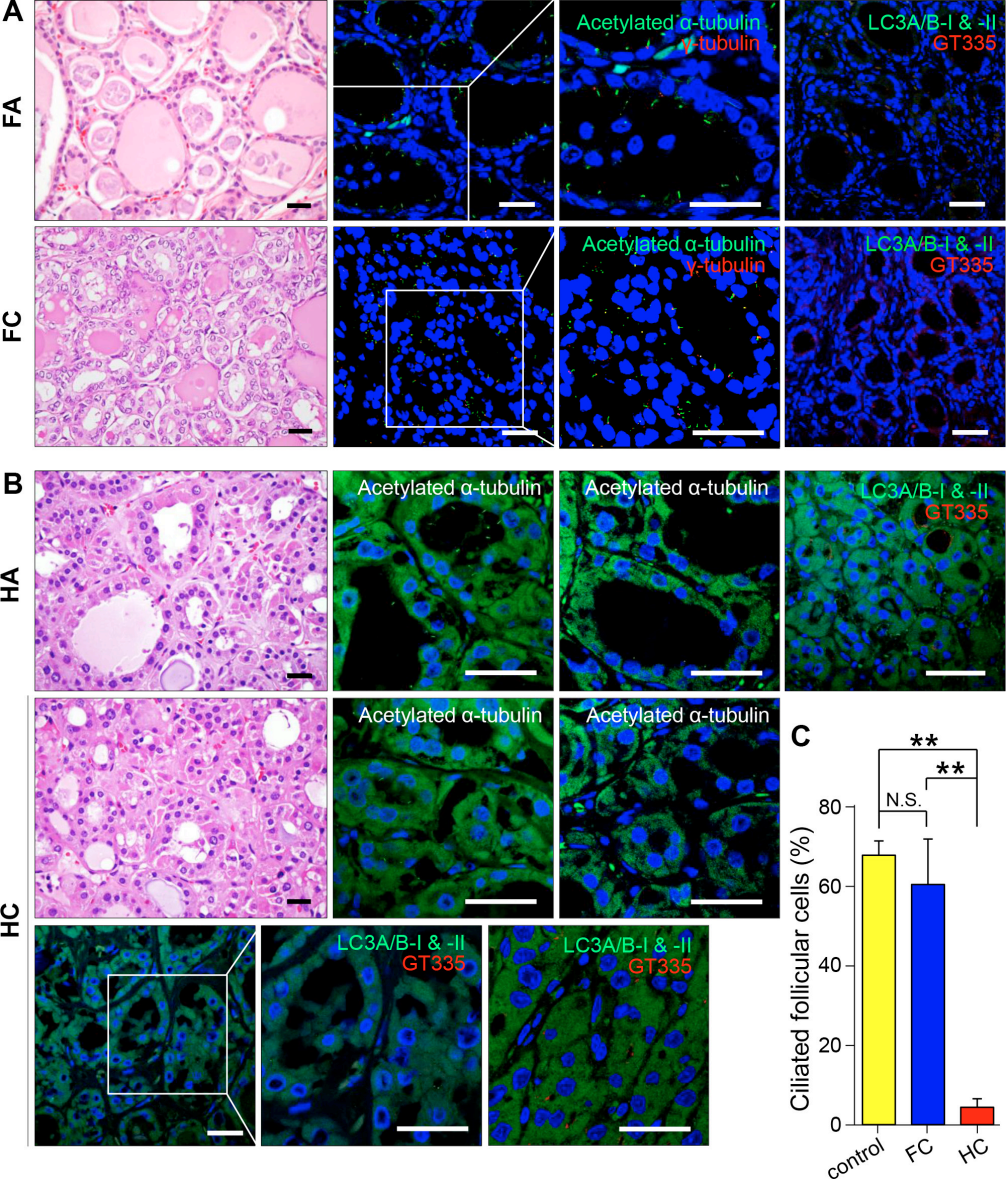
Distribution of primary cilia in follicular tumors and Hürthle cell tumors (**A**) Follicular adenoma stained with H&E. Immunofluorescent staining of primary cilia in tumorigenic follicular cells using anti-acetylated α-tubulin (green) and anti-γ-tubulin (red). Scale bar, 10 μm. (**B**) Hürthle cell adenoma stained with H&E. Immunofluorescent staining of primary cilia in tumorigenic Hürthle cells using anti-acetylated α-tubulin (green). Scale bar, 10 μm (first panels). Immunofluorescent staining of Hürthle cell tumors using anti-LC3A/B-I & II (green) and primary cilia using anti-acetylated α-tubulin (red). Scale bar, 10 μm (second, third, and fourth panels). (**C**) The average frequency of primary cilia in Hürthle cell carcinoma compared with that in normal thyroid (*p* = 0.0002) or follicular carcinoma (*p* = 0.008). Normal follicular cells were used as controls. **p < 0.01, N.S.; not significant.

A primary Hürthle cell tumor of the thyroid gland is a relatively rare type of differentiated thyroid cancer [26, 27]. It has a poorer clinical course than that of other differentiated thyroid cancers, and is considered a variant of the follicular tumor of the thyroid known as follicular carcinoma, oxyphilic type [26, 27]. However, several investigators contend that a Hürthle cell tumor is a distinct form of thyroid neoplasm differentiated from follicular neoplasms [26, 27]. We observed the formation of primary cilia in follicular adenomas (*n* = 10), follicular carcinomas (*n* = 10), Hürthle cell adenomas (*n* = 10) and Hürthle cell carcinomas (*n* = 10). As shown in Figure 4A, tumor cells of the follicular adenoma showed primary cilia in the apical membrane oriented toward the follicular lumen similar to normal thyroid follicles. By contrast, ciliogenesis was markedly decreased in Hürthle cell carcinomas (normal 67.8 ± 3.6% vs FC 60.5 ± 11.5% vs HC 4.4 ± 2.2%)(Figure 4B and 4C). Microtuble-associated protein light chain 3 (LC3) has been used as a specific marker for the monitoring of autophagy. As we previously reported, normal follicular cells and non-oncocytic cells of follicular tumors show rare expression of LC3, whereas oncocytes in Hürthle cell tumors consistently express high levels of LC3 [28]. Therefore, higher expression of LC3 has been used as a specific marker of Hürthle cells. As shown in Figure 4B, Hürthle cells that were positive for LC3 did not show primary cilia. It is likely that the suppression of ciliogenesis is a common feature of Hürthle cells, and the loss of primary cilia may contribute to the dysregulation of biological activities in Hürthle cells.

### Defective ciliogenesis in the Hürthle cell carcinoma cell line XTC.UC1

**Figure 5:**
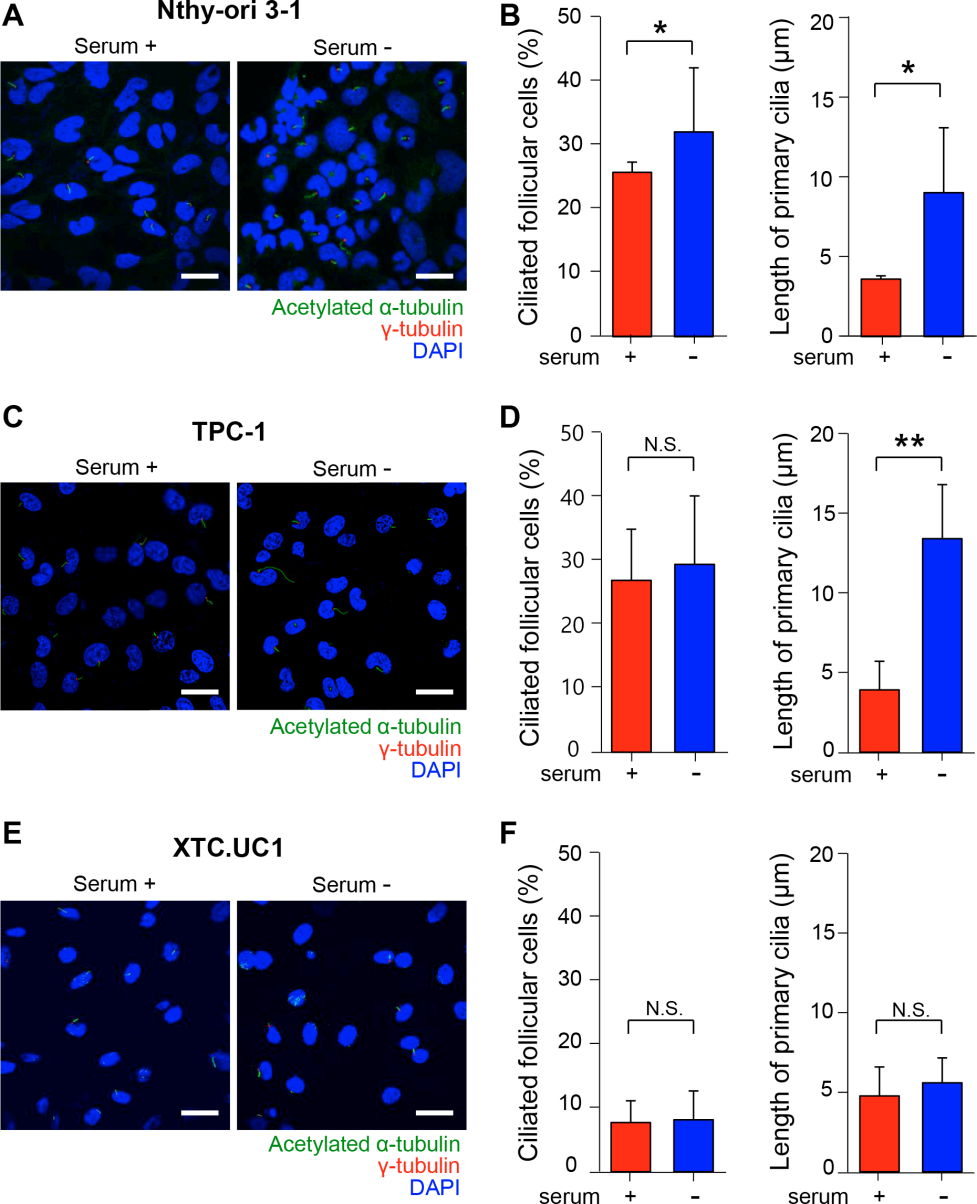
Primary cilia in human normal thyroid follicular cell lines and thyroid cancer cell lines (**A**, **C**, **E**) Immunofluorescent staining of primary cilia using anti-acetylated α-tubulin (green) and anti-γ-tubulin (red). Scale bar, 10 μm. (**B**, **D**, **F**) The average frequencies and the average lengths of primary cilia in Nthy-ori 3–1, TPC-1, and XTC.UC1 cells. serum +; serum-supplemented conditions, serum -; serum-starved conditions.

We examined primary cilia in Nthy-ori 3–1 cells, an untransformed human thyroid follicular cell line. Primary cilia were analyzed not only in the presence of serum but also in the absence of serum, because serum starvation is commonly used to induce ciliogenesis in cell culture [29–31]. Serum starvation induced a moderate increase in ciliated cell numbers in Nthy-ori 3–1 cells (Figure 5A). Approximately 26.2% of Nthy-ori 3–1 cells cultured in serum-supplemented conditions exhibited primary cilia, and approximately 32.6% of cells were ciliated after 36 hr of serum starvation (*p* = 0.037). Serum starvation clearly facilitated the elongation of primary cilia. The average length of primary cilia in Nthy-ori 3–1 cells was 3.62 ± 0.21 μm in serum-supplemented conditions and 9.06 ± 4.09 μm in serum-starved conditions for 36 hr (*p* = 0.014)(Figure 5B). To test whether ciliogenesis is affected by tumor transformation, we observed primary cilia in the thyroid cancer cell line TPC-1 cultured in the presence or absence of serum. The TPC-1 cell line was originally derived from a human papillary thyroid carcinoma containing rearrangements of the *RET* gene (RET/PTC). Although the difference in ciliated cell number was not statistically significant (serum-supplemented conditions 27.5 ± 1.6% vs serum-starved conditions 30.5 ± 10.2%, *p* = 0.43), serum starvation clearly facilitated the elongation of primary cilia (Figure 5C and 5D). The average length of the primary cilia was 4.97 ± 2.48 μm in serum-supplemented and 13.60 ± 4.23 μm in serum-starved conditions (*p* = 0.003)(Figure 5C and 5D).

We then assessed ciliogenesis in XTC.UC1 cells derived from Hürthle cell carcinoma. XTC.UC1 cells showed markedly decreased numbers of primary cilia compared with those of Nthy-ori 3–1 and TPC-1 cells (Figure 5). The frequency of primary cilia in XTC.UC1 cells was 7.75 ± 3.42% in serum-supplemented conditions and 8.19 ± 4.53% in serum-starved conditions (*p* = 0.74)(Figure 5E and 5F). Serum starvation-induced ciliary elongation was not observed in XTC.UC1 cells. The average length of the primary cilia was 4.77 ± 1.82 μm in serum-supplemented and 5.60 ± 1.57 μm in serum-starved conditions (*p* = 0.27) (Figure 5E and 5F). These results further support the idea that loss of cilia is a characteristic feature of Hürthle cells. It is likely that certain cellular factors that allow a thyrocyte to differentiate into a Hürthle cell may affect ciliogenesis.

### Pharmacological and genetic inhibition of autophagosome formation restores ciliogeneis in XTC.UC1 cells

**Figure 6:**
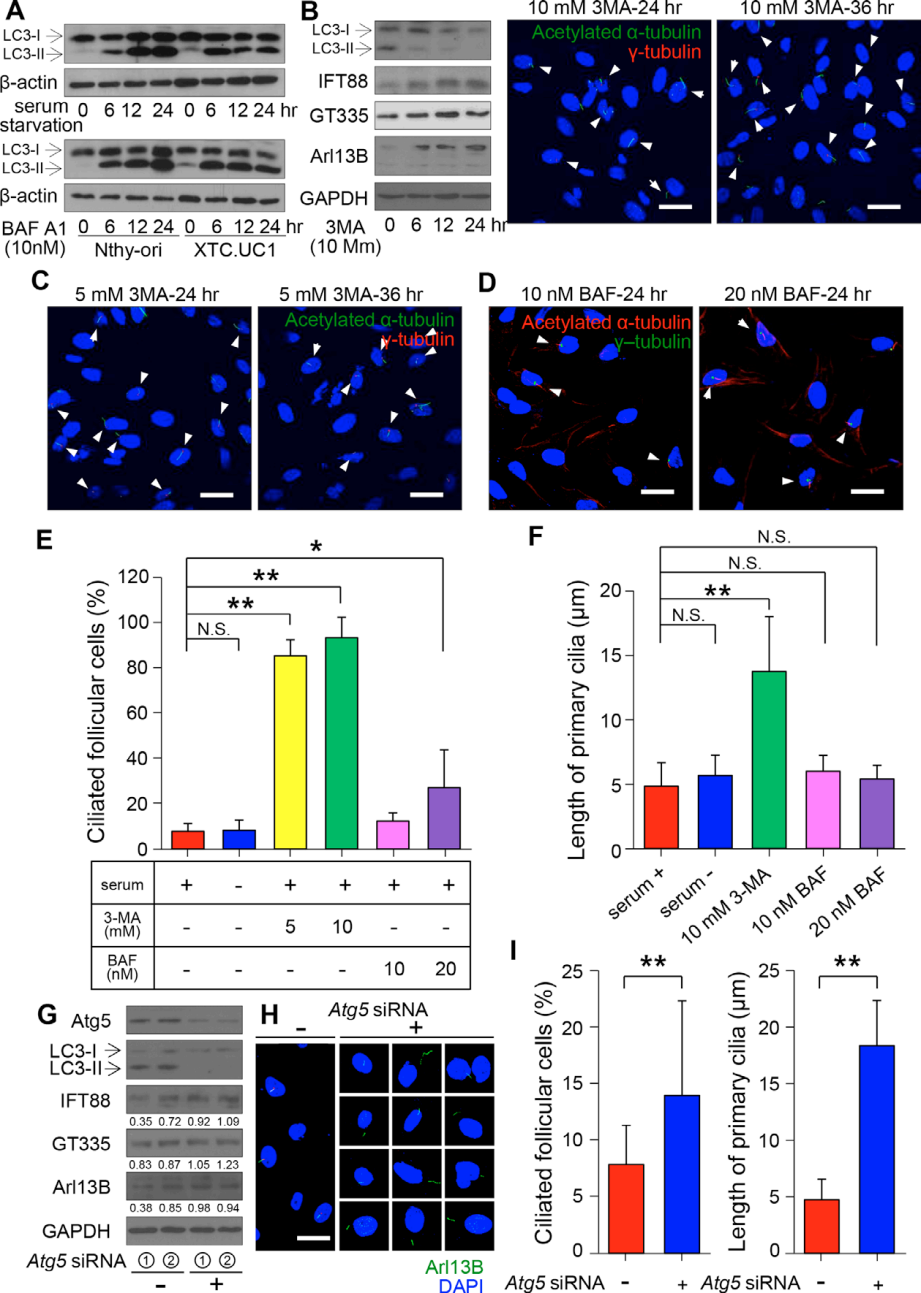
Relationship between primary ciliogenesis and autophagy in XTC.UC1 cells (**A**) Western blot analyses of LC3 levels in XTC.UC1 and Nthy-ori 3–1 cells after serum deprivation and BAF treatment. β-actin served as the loading control. (**B**) Western blot analyses of LC3, IFT88, ARL13B, and GT335 levels in XTC.UC1 after treatment with 10 mM 3-MA for 6, 12, or 24 hr. GAPDH served as the loading control. (**C**) Immunofluorescent staining of primary cilia (arrows) using anti-acetylated α-tubulin (green) and anti-γ-tubulin (red). Scale bar, 10 μm. (**D**) Immunofluorescent staining of primary cilia (arrows) using anti-acetylated α-tubulin (red) and anti-γ-tubulin (green). Scale bar, 10 μm. (**E**) The average frequency of primary cilia after the induction of autophagy by serum deprivation or its inhibition by 3-MA or BAF treatment. (**F**) The average length of primary cilia after the induction of autophagy by serum starvation or its inhibition by 3-MA or BAF treatment. *p < 0.05, **p < 0.01, N.S.; not significant. (**G**) Western blot analyses of ATG5, LC3, IFT88, GT335 and ARL13B expression in XTC.UC1 after transfection with *Atg5* siRNA. (**H**) Immunofluorescent staining of primary cilia of XTC.UC1 after transfection with *Atg5* siRNA using anti-Arl13B (green). Scale bar, 10 μm. (**I**) The average frequency and length of primary cilia after the inhibition of autophagy by *Atg5* siRNA. **p < 0.01.

Our previous study demonstrated that XTC.UC1 cells have higher basal autophagic flux, which can be further augmented by CCCP and bafilomycin A1 (BAF) treatment [12]. Consistently, we found a higher level of LC3-II under basal and BAF-treated conditions in XTC.UC1 cells than in Nthy-ori 3–1 cells (Figure 6A). To determine whether increased autophagic flux is associated with defective ciliogenesis in XTC.UC1 cells, we examined the frequency of ciliated cells as well as the length of cilia following treatment with inhibitors of phagopore and autophagolysosome formation. 3-MA inhibits autophagy by blocking autophagosome formation via the inhibition of class III phosphatidylinositol 3-kinases (PI3K) [32]. To test if increased autophagy in XTC.UC1 cells interferes with ciliogenesis by degrading proteins critical for ciliogenesis, we examined cilia expression in 3-MA-treated XTC.UC1 cells. As shown in Figure 6B, the levels of IFT88 and ARL13B, which regulate ciliogenesis, increased in response to 3-MA treatment. Interestingly, the frequency of ciliated cells was markedly higher with 3-MA treatment (10 mM, 89.2% ± 5.6%) than without treatment (8.1% ± 0.4%, *p* < 0.001)(Figure 6C and 5D). The lengths of the primary cilia were also significantly increased (13.6 μm for 3-MA *versus* 4.8 μm for the control, *p* = 0.0003)(Figure 6C and 6F). On the other hand, BAF prevents the maturation of autophagic vacuoles by inhibiting fusion between autophagosomes and lysosomes. BAF treatment also increased ciliated cell number, although its effect on ciliogenesis was milder than that of 3-MA. BAF treatment did not promote ciliary elongation (*p* = 0.27 with 10 nM BAF; *p* = 0.47 with 20 nM BAF) (Figure 6D, 6E and 6F). Although deprivation of serum increases LC3-II processing in XTC.UC1 cells, it did not further decrease ciliogenesis (Figure 6F). These observations indicate that ciliogenesis in the XTC.UC1 cell line is maintained at a maximally suppressed level that may be unresponsive to further activation of autophagic processes.

To further substantiate the role of autophagosome formation in the regulation of ciliogenesis, we used Atg5 siRNA to specifically inactivate the autophagosome in XTC.UC1 cells. *Atg5* siRNA achieved efficient knockdown of ATG5 protein expression and inhibited autophagic activity, as indicated by the decreased level of LC3-II (Figure 6G). The knockdown of ATG5 is normally able to prevent mitochondrial protein degradation. The levels of IFT88 and ARL13B increased in response to *Atg5* siRNA. Inhibition of autophagy or prevention of mitochondrial protein degradation induced ciliogenesis, as shown by the increases in the frequency of ciliated cells (13.8% ± 8.3%, *p* < 0.01) and in the lengths of primary cilia (18.5 ± 4.0 μm, *p* < 0.01) (Figure 6H and 6I) in XTC.UC1 cells.

**Figure 7:**
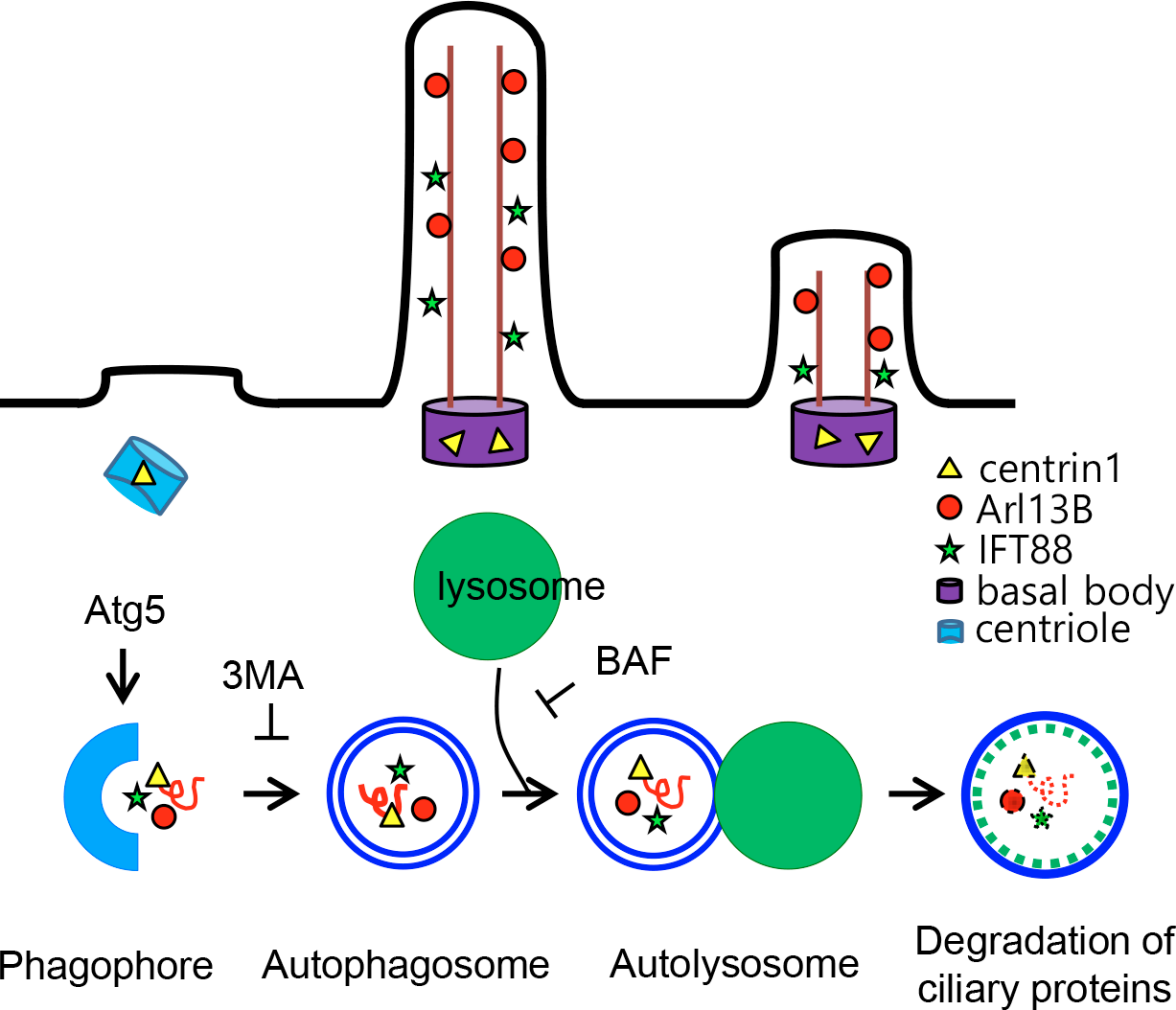
Schematic model for the regulation of ciliogenesis in XTC.UC1 Cells of the Hürthle cell carcinoma cell line, XTC.UC1, show high basal levels of autophagosome formation. 3-MA inhibits the cleavage of LC3-I and increases the levels of IFT88 and ARL13B by sequestrating structural proteins for ciliogenesis. Inhibition of autophagosome formation restores ciliogenesis via the accumulation of IFT88 and ARL13. Improved ciliogenesis is not observed in cells treated with BAF, an inhibitor of autophagosome fusion.

Taken together, these results suggest that ciliogenesis in the Hürthle cell cancer cell line, XTC.UC1, is negatively influenced by higher autophagic activity, a bona fide feature of this cell line. Defective ciliogenesis in Hürthle cells in benign and malignant diseases may be caused by persistent sequestration of ciliogeneic proteins, such as IFT88 and ARL13B (Figure 7).

## DISCUSSION

Primary cilia are thought to function as sensors of the follicular lumen environment, which plays a crucial role in maintaining follicular homeostasis. Although there is no clear evidence on the role of primary cilia in follicular homeostasis, patients with primary ciliopathy and animal models of defective ciliogenesis show profound hypothyroidism [33]. These observations indicate that abnormal ciliogenesis may be important for the development of thyroid diseases. In this study, we observed ciliogenesis by analyzing the frequency of ciliated cells and the lengths of cilia in the normal thyroid gland and in benign and malignant human thyroid diseases.

We found that a common benign thyroid disease, nodular hyperplasia, had no marked abnormalities in ciliogeneis compared with ciliogeneis in the normal thyroid. Hashimoto's thyroiditis is a representative chronic autoimmune thyroid disease characterized by the infiltration of immune cells and the presence of autoantibodies against thyroid autoantigens. We found the follicles in Hashimoto's thyroiditis to have a normal pattern of primary cilia. Patients with Hashimoto's thyroiditis examined in this study were euthyroid. Therefore, it is unclear how TSH regulates the ciliogenesis of thyroid epithelial cells *in vivo*. Immune cells and their production of multiple proinflammatory cytokines results in functional and structural abnormalities in thyroid epithelial cells. Hürthle cells found in Hashimoto's thyroiditis are thought to arise due to chronic inflammatory stress on epithelial cells. Therefore, markedly defective ciliogenesis in Hürthle cells of Hashimoto's thyroiditis may have been caused by inflammatory stress or defective oxidative metabolism in Hürthle cells.

Differentiated thyroid cancers such as PTC lose follicular structures because of the loss of epithelial cell polarity. However, we found that primary cilia were well preserved in PTC cells. Therefore, follicular structures are not the prerequisite structure for the expression of primary cilia in thyroid cancer. The clinical prognostic outcome of PTC is determined by several histological subclasses [34]. It was reported that the subtypes of the oncocytic, solid, and tall cell variants showed a more unfavorable clinical course than that of the conventional type of PTC [34]. The frequency of ciliated cells was not significantly different between histological subtypes, except for the oncocytic variant. Furthermore, the average length of cilia was also reduced in the oncocytic variant of PTC. These findings indicated that abnormal ciliogenesis was an inherent feature of Hürthle cells in inflammatory and tumorous thyroid diseases. The presence of Hürthle cells in the thyroid was attributed to impaired mitochondrial oxidative function caused by oxidative stress or a mutation in mtDNA [13]. Hürthle cell tumors are defined as being composed of at least 75% Hürthle cells [35]. Although several investigators propose that they are distinct from other follicular cell neoplasms [35], others consider them to be subtypes of follicular adenoma or carcinoma. Our results showed that Hürthle cells found in both primary and secondary thyroid lesions exhibited abnormal ciliogenesis.

It is interesting that abnormal ciliogenesis in Hürthle cell lesions was observed in both benign and malignant thyroid diseases. Because Hürthle cells display defective oxidative phosphorylation and increased basal autophagy that includes mitophagy, abnormal ciliogenesis may be associated with mitophagy or autophagy in Hürthle cells. The tumorigenic Hürthle cell line XTC.UC1 shows higher autophagosome formation; however, actual mitophagy turnover is lower because of dysfunctions in the regulation of PINK1 and Parkin-mediated mitophagy [12]. These molecular features might explain the inefficient clearance of abnormal mitochondria in Hürthle cell tumors. Recent studies show that autophagy and ciliogenesis are intricately linked. Tang *et al*. demonstrate that autophagy promotes ciliogenesis by degrading OFD1 at centriolar satellites [11]. By contrast, Pampliega *et al*. reveal that autophagy negatively regulates ciliogenesis by degrading the essential ciliary protein IFT20 [10]. In addition, Cuervo *et al*. reported that autophagy dysfunction can be attributed to impaired ciliary signaling [9]. The relationship between autophagy and primary cilia is bidirectionally regulated and specific to different cell types, environments, growth conditions, and stimuli. These observations indicate that the relationship between ciliogenesis and autophagy needs to be interpreted in the context of the specific disease. In this study, Hürthle cell tumors and XTC.UC1 cells showed high basal levels of autophagosome formation, and exhibited defects in primary ciliogenesis *in vitro* and *in vivo*. Upon formation, the autophagosome undergoes a stepwise maturation process that includes fusion events with the lysosome. Bafilomycin A1, a vacuolar H(+)-ATPase inhibitor, inhibits the fusion of the autophagosome with the lysosome. The ciliogenesis phenotype of XTC.UC1 cells was not affected by bafilomycin A1. Collectively, the data suggest that autophagosome formation, not lysosome fusion, is the critical step leading to defective ciliogenesis in XTC.UC1 cells. The findings that *Atg5* siRNA and 3-MA increased the levels of IFT88 and ARL13B in XTC.UC1 cells indicates sequestration of structural proteins for ciliogenesis during enhanced autophagosome formation (Figure 7). It is interesting that improved ciliogenesis was not observed in XTC.UC1 cells treated with BAF, which inhibits the fusion of the autophagosome, suggesting that the impairment of ciliogenesis in Hürthle cell tumors may affect an early stage of autophagy in the thyroid gland.

The population study based on the Surveillance, Epidemiology, and End Results (SEER) database has confirmed that Hürthle cell carcinoma is more aggressive and that patients with Hürthle cell carcinoma have a lower survival rate than those with other differentiated thyroid cancer subtypes [36]. Improved survival associated with small tumors confined to the thyroid without local and distant metastasis and with those treated with radioiodine therapy [36]. Ciliogenesis and autophagy are the determining factors in the prognosis of human cancers [11]. Abnormal ciliogenesis and increased autophagy correlate with poor prognosis in these specific forms of cancer [37]. Therefore, the impairment of ciliogenesis in Hürthle cell tumors may explain the relatively poor prognosis of differentiated thyroid cancers. In this study, we demonstrated that defects in the expression of genes involved in ciliogenesis is a hallmark of Hürthle cell tumors in the thyroid gland.

## MATERIALS AND METHODS

### Human thyroid specimens

Patients that underwent a thyroidectomy between January 2002 and December 2005 at St. Mary's Hospital, Daejeon, South Korea, were retrospectively enrolled. The baseline characteristics of each patient are summarized in Table 1. All patients presented with nodular hyperplasia of the thyroid, Hashimoto's thyroiditis, follicular adenoma and carcinoma, Hürthle cell adenoma and carcinoma, or PTC (conventional, follicular variant, oncocytic variant, and solid variant, tall cell variant). Contralateral normal tissues were obtained and used as controls. Two pathologists independently reviewed H&E-stained tissue cross-sections, and a diagnosis was made according to the World Health Organization classification of endocrine organ tumors [38]. The study protocol was reviewed and approved by the Institutional Review Board and the methods were carried out in accordance with the approved guidelines of the Daejeon St. Mary's Hospital, College of Medicine, the Catholic University of Korea. All participants provided signed, written informed consent.

**Table 1:** Summary of patients characteristics and clinical data

Diagnosis	Gender (*n*)		Age (yr)^a^	Tumor Size (cm)^a^
	Male	Female		
NH	0	10	51.2 (42–69)	1.4 (0.6–2.0)
HT	0	2	41.5 (37–46)	
FA	3	7	41.1 (26–68)	3.3 (1.0–9.0)
FC	2	8	48.6 (36–73)	3.5 (0.9–5.2)
HA	1	9	48.1 (41–58)	3.2 (1.0–7.0)
HC	1	9	51.5 (20–73)	3.8 (1.2–8.5)
PTC-conv	2	8	58.6 (32–73)	2.1 (1.0–7.0)
PTC-FV	0	10	50.2 (35–66)	1.9 (0.8–3.0)
PTC-SV	0	5	44.8 (20–73)	1.6 (0.8–3.3)
PTC-TV	0	5	55.8 (44–70)	1.9 (0.4–3.8)
PTC-OV	3	7	50.2 (34–77)	1.8 (1.0–4.0)

a Data represent the median and range.

Abbreviations: NH, nodular hyperplasia; HT, Hashimoto's thyroiditis; FA, follicular adenoma; FC, follicular carcinoma; HA, Hürthle cell adenoma; HC, Hürthle cell carcinoma; PTC-conv, conventional; PTC-FV, follicular variant; PTC-SV, solid variant; PTC-TV, tall cell variant; PTC-OV, oncocytic variant of papillary carcinoma.

### Cell lines, culture conditions, and chemicals

The Hürthle cell carcinoma cell line XTC.UC1 and TPC-1 cells were cultured in Dulbecco's Modified Eagle medium (Gibco^®^) supplemented with 5% fetal bovine serum (FBS), 100 U/ml penicillin, and 100 μg/ml streptomycin at 37°C in a humidified atmosphere of 5% CO_2_ [39]. The untransformed human thyroid cell line Nthy-ori 3–1 was provided by the European Collection of Authenticated Cell Cultures and maintained in RPMI 1640 medium (Gibco^®^) supplemented with 5% FBS, 100 U/ml penicillin, and 100 μg/ml streptomycin at 37°C in a humidified atmosphere of 5% CO_2_.

### Immunofluorescence staining

Paraffin-embedded 7 μm-thick tissue cross-sections were placed in an oven and incubated at 56°C for 3 hr. Thereafter, cross-sections were deparaffinized in xylene and rehydrated through a graded-series of ethanol baths. Antigens were retrieved in antigen retrieval buffer (0.01 M citric acid–sodium citrate, pH 6.0) by heating the cross-sections in an autoclave at 121°C for 25 min. After washing, the cross-sections were air-dried for 30 min and then re-washed with 1 × phosphate-buffered saline (PBS, 10 mM Na_2_HPO_4_, pH 7.4 and 150 mM NaCl). Cells were cultured on round coverslips in 12-well plates for 48 hr after seeding. After incubation under the indicated conditions, the cells were washed with 1 × PBS. The tissue cross-sections and cultured cells were fixed with 4% paraformaldehyde in PBS for 15 min, and then permeabilized with 0.5% Triton X-100 in PBS for 10 min at room temperature. Permeabilized cells were blocked with 5% bovine serum albumin in PBS for 30 min at room temperature. Thereafter, tissue cross-sections and cultured cells were incubated with primary antibodies for 24 hr at 4°C. On the following day, the slides and coverslips were washed three times with 1 × PBS and incubated at 4°C for 12 hr with secondary antibodies. Primary antibodies against LC3 (Sigma-Aldrich), acetylated α-tubulin (Sigma-Aldrich), Arl13B (ProteinTech Group), and γ-tubulin (Sigma-Aldrich) were used. Goat anti-mouse and goat anti-rabbit secondary antibodies conjugated to Alexa Fluor dyes (Invitrogen/Life Technologies) were used for indirect fluorescent detection. The stained slides were observed under an Olympus FluoView FV1000 microscope equipped with a charge-coupled device camera (Olympus Corp.).

### Analysis of cilia frequency in cell lines and thyroid tissue

The frequency of ciliated cells in cultures and thyroid tissue was determined by counting acetylated α-tubulin- or Arl13B-positive cilia in 1000 cell nuclei. We determined the frequency of ciliated cells in human thyroid tissues by the following method. We prepared five paraffin blocks with control and diseased tissues, and prepared two slides from each paraffin block. Cross-sections were immunostained with the indicated antibodies and 1000 follicles were inspected. Primary cilia were manually counted. Primary cilia length was measured using the length measurement tool within the software package (Olympus Corp.).

### Detection of primary cilia after inhibition of autophagosome formation

To silence the function of ATG5, XTC.UC1 cells were transfected with *Atg5* siRNA (100 nM) using Lipofectamine^®^ RNAiMAX (Invitrogenm Carlsbad, CA, USA) according to the manufacturer's protocol. The medium was replaced after 6 hr and cells were incubated for a further 48 hr. Knockdown of ATG5 was confirmed for each experiment by performing western blot analysis with anti-ATG5 antibody (Cell signaling). XTC.UC1 cells were cultured on coverslips in 12-well plates for 48 hr after seeding. Thereafter, cells were treated with 5 mM and 10 mM 3-methyladenine (3-MA; Sigma-Aldrich) or 10 and 20 nM bafilomycin A1 (BAF; Sigma-Aldrich) for 0, 12, 24, and 36 hr. After immunofluorescent staining, the stained slides were observed under a confocal microscope (Olympus Corp.).

### Western blot analysis

Cells were washed twice with PBS and lysed in RIPA lysis buffer (10 mM Tris-HCl, pH 8.0, 150 mM NaCl, and 1% Nonidet P-40) supplemented with a broad-spectrum protease inhibitor cocktail (Roche). Protein concentrations were measured using the Bradford assay. Proteins were denatured by boiling for 5 min. Samples were resolved by 10% SDS-PAGE and transferred to Hybond ECL membranes (Amersham Pharmacia Biotech). The membranes were blocked for 30 min in Tris-buffered saline containing 0.1% Tween 20 (TBS/T) and 5% non-fat milk, and then incubated overnight at 4°C with primary antibodies against LC3 (Sigma-Aldrich), IFT88 (ProteinTech Group), Arl13B (ProteinTech Group), Polyglutamylation Modification (GT335, AdipoGen), and GAPDH (Abcam). The membranes were washed three times with TBS/T and incubated with a horseradish peroxidase (HRP)-conjugated secondary antibody (Phototope-HRP Western blot detection Kit; New England Biolabs) for 2 hr at room temperature. After three washes for 10 min each, the blots were developed using the LumiGLO chemiluminescent substrate (Cell Signaling Technology).
